# Patient and clinician satisfaction with home parenteral nutrition intestinal failure telemedicine consultations: A survey of clinicians and patients

**DOI:** 10.1002/ncp.11280

**Published:** 2025-02-12

**Authors:** Ayodele Sasegbon, Amy Woods, Francesca Vourloitis, Maria Barrett, Cristina Cuerda, Palle B. Jeppesen, Francisca Joly, Georg Lamprecht, Manpreet Mundi, Kinga Szczepanek, Andre Van Gossum, Tim Vanuytsel, Geert Wanten, Loris Pironi, Simon Lal

**Affiliations:** ^1^ Intestinal Failure Unit, Salford Royal Hospital Salford UK; ^2^ University of Manchester Manchester UK; ^3^ Departamento de Medicina Universidad Complutense de Madrid, Nutrition Unit, Hospital General Universitario Gregorio Marañón Madrid Spain; ^4^ Department of Gastroenterology Rigshospitalet Copenhagen Denmark; ^5^ Department of Gastroenterology and Nutritional Support, Center for Intestinal Failure, Reference Centre of Rare Disease MarDI, AP‐HP Beaujon Hospital University of Paris Inserm UMR Paris France; ^6^ Department of Medicine II, Division of Gastroenterology and Endocrinology Rostock University Medical Center Rostock Germany; ^7^ Division of Endocrinology, Diabetes, Metabolism, and Nutrition Mayo Clinic College of Medicine Rochester Minneapolis USA; ^8^ General Surgery Unit with Intestinal Failure Center, Stanley Dudrick's Memorial Hospital Skawina Poland; ^9^ Medico‐Surgical Department of Gastroenterology, Hopital Erasme Free University of Brussels Brussels Belgium; ^10^ Translational Research Center for Gastrointestinal Disorders, KU Leuven Leuven Belgium; ^11^ Department of Medical and Surgical Sciences University of Bologna Bologna Italy; ^12^ Centre for Chronic Intestinal Failure, IRCCS AOUBO Bologna Italy

**Keywords:** consultations, intestinal failure, satisfaction, telemedicine, telephone, video

## Abstract

**Introduction:**

Despite the widespread use of telemedicine to assess patients with intestinal failure (IF) receiving home parenteral nutrition (HPN), satisfaction with remote consultation methods has not been comprehensively assessed. Here, we assessed patient and clinician attitudes to telephone and video IF consultations.

**Methods:**

Telemedicine questionnaires were designed and distributed in paper form to patients with IF receiving HPN under the care of a UK national IF reference center and electronically to IF clinicians in Europe, North America, Australia, and New Zealand.

**Results:**

Seventy‐eight patients (53 women and 25 men) and 110 clinicians agreed to complete the questionnaires. Sixty seven percent of patients who had telephone consultations and 50% of patients who had video consultations were satisfied with their consultations. Forty nine percent and 83% of patients who had telephone and video consultations, respectively, felt they were of the same standard as their face‐to‐face consultations. Despite 60% of clinicians feeling telemedicine training would be useful, 55% of clinicians stated they mostly or always met all of the needs of their patients via telephone consultations, whereas 96% of clinicians felt similarly for video consultations (*P* = 0.002). A total of 33% and 57% of clinicians felt telephone and video consultations, respectively, were of the same standard as face‐to‐face consultations (*P* = 0.004).

**Conclusion:**

This study comprehensively assesses attitudes to IF telemedicine consultations. Our data show that a large proportion of patients and clinicians are satisfied with IF telephone and video consultations. However, there is an unmet need amongst clinicians for telemedicine training.

## BACKGROUND

Intestinal failure (IF) can be defined as the inability of an individual's gastrointestinal tract to absorb the fluids, as well as micro and macronutrients, required to maintain their physiological processes and keep them in good health.^1^ Patients with type 3 IF require long‐term home parenteral nutrition (HPN) and regular input from multidisciplinary clinical teams to optimize their care.^1^ The provision of this therapeutic oversight and support necessitates periodic clinic appointments.^1^

The onset of the coronavirus disease 2019 (COVID‐19) pandemic and the infection control measures that were introduced to prevent viral spread prompted an urgent reassessment of the traditional face‐to‐face method of seeing patients routinely in outpatient departments.^2^, ^3^ Video and telephone consultations, collectively known as telemedicine, were used more frequently to ensure patients could be regularly reviewed while protecting patients and clinicians from the spread of infection.^2^, ^4^, ^5^ After the global use of COVID‐19 vaccines and the reduced prevalence of the virus, telemedicine has been posited as a means of addressing the multiple pandemic‐induced backlogs that have built up in health systems around the world.^2^, ^4^ This is in spite of mixed evidence of the time‐saving benefits of telemedicine.^6^, ^7^

Since the pandemic, there have been attempts to assess the attitudes of clinicians towards telemedicine.^8^ To this end, clinician surveys have been conducted in multiple areas of medicine, including gastroenterology.^9^, ^10^, ^11^ These have consistently shown high levels of satisfaction with both telephone and video consultations.^10^, ^11^ However, no studies of IF clinician attitudes to telemedicine have been published. Similarly, regarding patient attitudes to telemedicine, studies in other areas of medicine have shown that patients are broadly satisfied with this remote approach to their care.^11^, ^12^ A single patient telemedicine satisfaction study has been performed in IF.^13^ In 2020, Cloutier et al demonstrated a mean telemedicine satisfaction of 85% in their cohort of 25 patients.^13^ However, in the study, the authors focused on satisfaction with video consultations. No study has assessed the satisfaction of patients with IF towards telephone consultations.

The views of patients with IF, and the clinicians who care for them, regarding telephone and video consultations in the current post COVID‐19 pandemic health landscape, constitute gaps in our knowledge that must be addressed.

## AIMS

1. To assess the attitudes of patients with IF to HPN telephone and video consultations in a UK national IF center.
2. To assess international IF clinician attitudes towards telephone and video consultations.

## METHODS

### Survey design

Two anonymous questionnaires were designed, one for patients with IF receiving HPN and one for clinicians who care for patients with IF receiving HPN. The patient questionnaire was developed by a multidisciplinary team of clinicians from a UK national IF reference center with input from patients. The clinician questionnaire was developed in collaboration with clinicians from the European Society for Clinical Nutrition and Metabolism (ESPEN) Chronic Intestinal Failure Special Interest Group.

The patient questionnaire was approved by the Northern Care Alliance National Health Service (NHS) Trust as a Service Improvement Project (reference No. 22HIP48). Confidentiality and anonymity were maintained at all times with only members of the IF clinical team being able to interact with patients. Verbal consent was obtained from all patients. Because patient questionnaires were entirely anonymous, non hypothesis driven, non interventional, and involved service evaluation, the study did not require ethical approval. This was verified by the University of Manchester research ethics tool.

The patient questionnaire had 28 items that evaluated attitudes toward both telephone and video consultations. It was composed of four interlinked subsections:

1. demographics;
2. attitudes towards telephone consultations;
3. attitudes towards video consultations;
4. accessibility.

The clinician questionnaire was composed of 73 items. Similarly to the patient questionnaire, it evaluated attitudes to telephone and video consultations, and was broadly divided into four subsections:

1. demographics;
2. attitudes towards telephone consultations;
3. attitudes towards video consultations;
4. assessment and management of patients.

It was understood while developing both the patient and clinician questionnaires that all questions may not apply to all survey participants. Both questionnaires were designed using the survey tool Qualtrics XM.

### Distribution

The patient questionnaire was circulated to patients with IF in three ways. Patients were approached: when attending their outpatient appointments, on the IF ward, or via telephone. Interested patients had the choice of completing the questionnaire either on paper, verbally over the telephone, or digitally via a Qualtrics weblink.

For the clinician questionnaire, a Qualtrics generated weblink was circulated electronically by the British Association of Parenteral and Enteral Nutrition (BAPEN) and European Society of Clinical Nutrition and Metabolism (ESPEN).

### Data analysis

Data from both the patient and clinician questionnaires were analyzed arithmetically and presented as proportions and percentages. Differences in attitudinal response patterns between sexes and age (≥65 years of age vs ≤64 years of age) in the patient questionnaire and geographic location of clinical practice in the clinician questionnaire were displayed and statistically analyzed, where appropriate, using the *χ* ^2^ test and Student *T* test. All analyses were performed using Qualtrics.

## RESULTS

### Patient Study

#### Demographics

Seventy‐nine patients with IF agreed to participate in the study. Four others who were approached declined to participate with no reason given, leading to a response rate of 95% (79/83). One patient had not had a telemedicine consultation and was subsequently excluded (*n* = 78). A total of 32% (*n* = 25) of participants were male, whereas 68% (*n* = 53) were female. The mean age of participants was 56 years (SD: 16).

#### Consultations in general

Ninety percent of participants indicated they were satisfied or very satisfied with face‐to‐face consultations. Most telemedicine was delivered via the telephone with 90% (*n* = 70) of participants having solely received telephone IF clinic assessments. Ten percent (*n* = 8) of participants had received both telephone and video consultations. No participant had received telemedicine consultations solely via video, which resulted in a low representation of video consultations.

#### Telephone consultations

Focusing on patients who expressed agreement or strong agreement, most patients (84%) stated they were comfortable speaking to health professionals over the phone (Table 1). Eighty‐eight percent of patients were able to hear their health professionals clearly, with only 5% of participants disagreeing with the statement. The majority of patients (90%) felt their telephone consultations were private, and 83% did not feel rushed. Eighty‐seven percent of patients felt they were able to ask all the questions they wanted to and discuss things that they felt were important. However, less than half (49%) of patients felt their telephone consultations were of the same standard as their face‐to‐face consultations. Despite this, most patients (67%) expressed a high degree of satisfaction with telephone consultations and felt telephone consultations were an acceptable way to interact with IF health professionals (74%).

**Table 1:** HPN IF patient attitudes towards telephone consultations (sex analysis).

	*N*	Strongly disagree, %	Disagree, %	Neither agree nor disagree, %	Agree, %	Strongly agree, %	*P* value
I feel comfortable talking to health professionals over the phone							
Total	77	0	10	6	55	29	–
Female	53	0	13	10	51	26	0.24
Male	24	0	4	0	63	33	
I could hear my health professional clearly							
Total	77	0	5	6	55	34	–
Female	53	0	6	9	55	30	0.40
Male	24	0	4	0	54	42	
I felt my conversation was private							
Total	77	0	1	9	59	31	–
Female	53	0	2	6	64	28	0.26
Male	24	0	0	17	46	37	
My consultation was of the same standard as my face‐to‐face consultations							
Total	75	1	33	16	32	17	–
Female	51	2	33	20	29	16	0.67
Male	24	0	33	8	38	21	
I did not feel rushed							
Total	76	1	5	11	53	30	–
Female	52	2	6	8	56	28	0.70
Male	24	0	4	17	46	33	
I was able to ask questions and discuss things that are important to me							
Total	76	1	7	5	58	29	–
Female	52	2	6	4	59	29	0.86
Male	24	0	8	9	54	29	
I am satisfied with telephone consultations							
Total	76	3	8	22	42	25	–
Female	52	2	8	25	40	25	0.92
Male	24	4	8	17	46	25	

Abbreviations: HPN, home parenteral nutrition; IF, intestinal failure.

The majority of participants (69%) indicated that appropriate adjustments such as flexible scheduling of their consultations and the provision of interpreters could be made to their telephone consultations to facilitate their participation. Ninety‐three percent of patients did not experience any technical difficulties during their telephone consultations. Of the 7% of patients who did experience technical difficulties, most indicated that this was due to poor mobile phone connectivity.

#### Video consultations

The majority of patients expressed that they could hear and see health professionals clearly (86%) (Table 2). However, only 57% expressed agreement or strong agreement with the statements that they felt comfortable during video consultations with a similar proportion feeling their conversations were private. No patient felt rushed during their video consultations. Seventy‐one percent of patients felt they were able to ask questions and discuss things that were important to them. Eighty‐three percent of patients felt that the quality of their video consultations were of the same standard as their face‐to‐face consultations. Additionally, 83% of patients were satisfied with video consultations, whereas 67% felt video consultations were an acceptable way to interact with IF health professionals.

**Table 2:** HPN IF patient attitudes towards video consultations.

	*N*	Strongly disagree, %	Disagree, %	Neither agree nor disagree, %	Agree, %	Strongly agree, %
I feel comfortable talking to health professionals over the phone	7	14	14	15	43	14
I could hear and see my health professional clearly	7	0	14	0	57	29
I felt my conversation was private	7	14	14	14	29	29
My consultation was of the same standard as my face‐to‐face consultations	6	0	17	0	50	33
I did not feel rushed	7	0	0	14	43	43
I was able to ask questions and discuss things that are important to me	7	0	14	14	29	43
How satisfied are you with video consultations	6	17	0	33	33	17

Abbreviations: HPN, home parenteral nutrition; IF, intestinal failure.

Three participants responded to the question about adjustments being made to facilitate their video consultations, with most (67%) indicating that their needs were accommodated. No patient experienced any technical difficulties during video consultations.

#### Accessibility

The mean one‐way distance from patients' homes to the IF center was 29 miles (SD: 26), with a range of 0.5–100 miles. Thirty‐six percent of patients agreed or strongly agreed with the statement that telemedicine consultations better fit into their day‐to‐day lives. This was similar to the 37% of patients who were neutral and greater than the 27% of patients who disagreed. Interestingly, 61% of patients stated that telemedicine consultations made them feel safer with respect to the ongoing, albeit reduced, risk of contracting COVID‐19.

When asked about aspects of telemedicine consultations they enjoyed, most patients stated that they were convenient. Conversely, when patients were asked how telemedicine consultations could be improved, some patients suggested giving patients the option to call clinicians back if they thought of anything else they wanted to raise. Forty‐three percent of patients indicated they would prefer their future consultations to be delivered in a hybrid manner, combining telemedicine and face‐to‐face consultations. Twelve percent of patients stated that they wanted future consultations to be delivered via telemedicine alone, whereas 45% of patients only wanted face‐to‐face consultations.

#### Sex subanalyses

No significant differences emerged between the responses of men and women for telephone consultations (Table 1). A similar statistical analysis was not performed for patients who had received video consultations because of the small number of patients who had been assessed in that manner (Table 2).

#### Age subanalyses

No significant differences emerged between the attitudes of patients older or younger than 65 years of age (Table 3). Because of the small number of patients who had video consultations, no age subanalysis was performed for video consultations.

**Table 3:** HPN IF patient attitudes towards telephone consultations (age analysis).

	*N*	Strongly disagree (%)	Disagree (%)	Neither agree nor disagree (%)	Agree (%)	Strongly agree (%)	*P* value
I feel comfortable talking to health professionals over the phone							
Total	77	0	10	6	55	29	–
≥65 years	30	0	17	6	50	27	0.06
<65 years	47	0	6	7	57	30	
I could hear my health professional clearly							
Total	77	0	5	6	55	34	–
≥65 years	30	0	10	10	50	30	0.46
<65 years	47	0	2	4	57	36	
I felt my conversation was private							
Total	77	0	1	9	59	31	–
≥65 years	30	0	3	7	63	27	0.76
<65 years	47	0	0	11	55	34	
My consultation was of the same standard as my face‐to‐face consultations							
Total	75	1	33	16	32	17	–
≥65 years	29	0	45	17	28	10	0.47
<65 years	46	2	26	15	35	22	
I did not feel rushed							
Total	76	1	5	11	53	30	–
≥65 years	30	0	3	13	50	34	0.91
<65 years	46	2	7	9	54	28	
I was able to ask questions and discuss things that are important to me							
Total	76	1	7	5	58	29	–
≥65 years	30	0	3	10	60	27	0.55
<65 years	46	2	30	2	9	57	
I am satisfied with telephone consultations							
Total	76	3	8	22	42	25	–
≥65 years	30	3	13	17	47	20	0.49
<65 years	46	2	5	26	39	28	

Abbreviations: HPN, home parenteral nutrition; IF, intestinal failure.

### Clinician study

#### Demographics

One hundred and fourteen clinicians accessed the online survey using the Qualtrics link provided. Of these, 110 consented to complete the questionnaire, a response rate of 97%. Ninety‐five clinicians completed at least three questions. Most clinicians who took part were medical doctors (53%). Other professions included dietitians (23%), nurses (15%), pharmacists (6%), psychologists (1%), and stoma therapists (1%). The number of patients with IF under the care of survey clinicians ranged from 1 to 400 with a mean of 98 and SD of 117. Clinicians had been qualified for a mean of 18 years (SD: 11) with a range of 1–40 years.

The majority of clinicians (94%) worked in university hospitals. Seventy percent of clinicians worked in Europe. In more detail, 33% of clinicians worked in the United Kingdom and 37% worked in continental Europe. A total of 21% of clinicians worked in Australia or New Zealand, and 9% worked in North America.

#### Consultations in general

A total of 90% of clinicians assessed patients with IF receiving HPN face‐to‐face in clinics, whereas 76% of clinicians performed telemedicine consultations. Of those who indicated that they performed telemedicine consultations, 55% performed telephone consultations, 7% performed video consultations, and 38% performed both. Most clinicians performed more face‐to‐face consultations than telemedicine consultations, with 59% stating that ≥75% of their consultations were face‐to‐face (Table 4).

**Table 4:** Clinicians' proportion of telemedicine and face‐to‐face consultations.

	*N*	>75% telemedicine, %	75% telemedicine and 25% face‐to‐face, %	50% telemedicine and 50% face‐to‐face, %	25% telemedicine and 75% face‐to‐face, %	>75% face‐to‐face, %
Can you estimate your proportional split of face‐to‐face and telemedicine consultations for patients with IF in your current practice?						
Total	55	2	11	29	29	30
Europe	39	3	5	30	31	31
Australia/NZ	11	0	37	27	18	18
North America	5	0	0	20	20	60
Going forward, what proportional split between telemedicine and face‐to‐face consultations does your IF service intend to implement?						
Total	51	0	21	48	25	6
Europe	36	0	11	58	22	8
Australia/NZ	11	0	64	27	9	0
North America	4	0	0	25	75	0

Abbreviations: HPN, home parenteral nutrition; IF, intestinal failure; NZ, New Zealand.

Most clinicians (95%) who performed telemedicine consultations had no formal telemedicine training, with 30% indicating that they would like formal training, and 46% indicating that, although they do not feel they require training at present, they would have liked to be offered training when they first started consulting with patients using telemedicine. Twenty‐three percent of clinicians did not want telemedicine training. When asked which telemedicine modality they preferred, 63% of clinicians indicated that they had no preference, whereas 27% preferred video consultations and 10% preferred telephone consultations.

Most clinicians indicated that their last face‐to‐face (88%) and telemedicine consultations (77%) were <1 month before completing the questionnaire (Table 5). Twenty‐eight percent of clinicians said that, after the introduction of telemedicine consultations, there had been a reduction in allocated IF clinic space within their hospitals. The vast majority of those clinicians (92%) were somewhat or very dissatisfied with the reduction, with one clinician stating that they felt increased pressure to see more patients more quickly since the introduction of telemedicine consultations.

**Table 5:** Timing of clinicians’ most recent telemedicine and face‐to‐face HPN IF appointments.

	Face‐to‐face, %	Telemedicine, %
<1 month ago	88	77
1–4 months ago	9	20
4–6 months ago	2	3
>6 months ago	1	0

Abbreviations: HPN, home parenteral nutrition; IF, intestinal failure.

#### Telephone consultations

The majority of clinicians (71%) agreed or strongly agreed that they were able to sustain good therapeutic relationships with their patients during telephone IF consultations and that telephone consultations were appropriately private (78%) (Table 6). However, one‐third of clinicians (33%) felt that telephone consultations were of the same standard as face‐to‐face consultations. Similarly, only 55% of participants stated they were able to meet the needs of their patients over the telephone most or all of the time (Table 7). When asked to explain why, the most common reason given was being unable to physically examine patients.

**Table 6:** Clinician attitudes towards telephone and video HPN IF consultations (geographic analysis).

	*N*	Strongly disagree (%)	Disagree (%)	Neither agree nor disagree (%)	Agree (%)	Strongly agree (%)	*P* value
Telephone, “I feel able to sustain a good therapeutic relationship with my patients during telephone consultations”							0.09
Total	79	0	10	19	45	26	
Europe	56	0	11	14	45	30	
Australia/NZ	15	0	7	33	40	20	
North America	8	0	12	25	63	0	
Video, “I feel able to sustain a good therapeutic relationship with my patients during video consultations”							
Total	27	0	0	7	50	43	
Europe	21	0	0	5	43	52	
Australia/NZ	5	0	0	20	60	20	
North America	1	0	0	0	100	0	
Telephone, “It is possible to maintain confidentiality/talk in confidence during telephone consultations”							0.33
Total	79	0	3	20	43	35	
Europe	56	0	3	18	41	38	
Australia/NZ	15	0	0	40	40	20	
North America	8	0	0	0	63	38	
Video, “It is possible to maintain confidentiality/talk in confidence during video consultations”							
Total	277	0	0	7	54	39	
Europe	21	0	0	4	48	48	
Australia/NZ	5	0	0	0	80	20	
North America	1	0	0	0	100	0	
Telephone, “The clinical care provided over the telephone is at the same standard as is usually provided during face‐to‐face consultations”							
Total	79	8	36	24	29	4	0.004*
Europe	56	7	36	23	29	5	
Australia/NZ	15	6	27	27	40	0	
North America	8	12	50	25	13	0	
Video, “The clinical care provided during video consultations is of the same standard as is usually provided during face‐to‐face consultations”							
Total	27	0	7	36	39	18	
Europe	21	0	0	28	48	24	
Australia/NZ	5	0	40	40	20	0	
North America	1	0	0	100	0	0	

* Statistical significance P value.

Abbreviations: HPN, home parenteral nutrition; IF, intestinal failure; NZ, New Zealand.

**Table 7:** Clinician's perceived ability to meet their HPN IF patients' needs using telephone and video consultations (geographic analysis).

	*N*	Never (0%), %	Not usually (25%), %	Sometimes (50%), %	Most of the time (75%), %	Always (100%), %	*P* value
Telephone, “I am able to meet all the needs of my patients via telephone consultations”							0.002
Total	65	1	9	35	55	0	
Europe	42	2	12	31	55	0	
Australia/NZ	15	0	7	40	53	0	
North America	8	0	0	37	63	0	
Video, I am able to meet all the needs of my patients via video consultations							
Total	2279	0	0	4	87	9	
Europe	16	0	0	0	87	13	
Australia/NZ	5	0	0	20	80	0	
North America	1	0	0	0	100	0	

Abbreviations: HPN, home parenteral nutrition; IF, intestinal failure; NZ, New Zealand.

Sixty‐eight percent of clinicians felt they were able to make reasonable adjustments to their telephone consultations to ensure they were accessible for their patients. Ninety‐four percent of clinicians stated that patients were always or mostly available to speak at the scheduled appointment time.

#### Video consultations

Most clinicians (93%) felt able to sustain good therapeutic relationships with their patients during video consultations (Table 6). Ninety‐three percent of clinicians also felt video consultations were private. Just over half (57%) of clinicians felt video consultations were of the same standard as face‐to‐face consultations. Significantly more clinicians (*P* = 0.004) felt that video consultations were of the same standard as face‐to‐face consultations (57%) than felt that telephone consultations were of the same standard (34%) (Table 6). Ninety‐six percent of clinicians felt they were able to meet the needs of their patients ≥75% of the time (Table 7). Comparing clinician responses between telephone and video consultations revealed a significant difference between telemedicine modalities with more clinicians stating they were able to meet most or all their patients’ needs using video consultations (*P* = 0.002). The most common reason preventing clinicians from meeting all of the needs of their patients was being unable to physically examine patients.

Most clinicians (82%) felt able to make reasonable adjustments to facilitate their patients’ access to video consultations. Lastly, 92% of clinicians felt their patients were mostly or always available to speak at their scheduled appointment time.

#### Assessment and management

**Figure 1:**
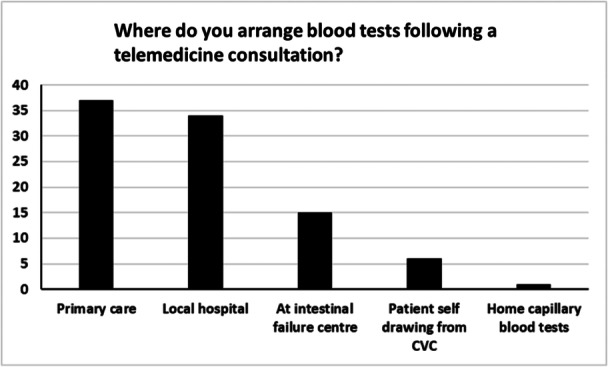
Graph showing the number of clinicians and where they arrange needed blood tests after telemedicine consultations. CVC, central venous catheter.

**Figure 2:**
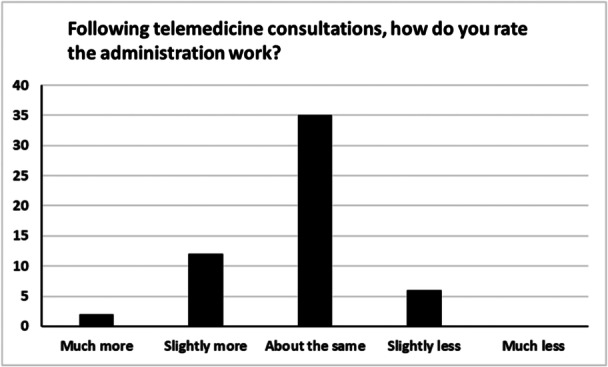
Graph showing the number of clinicians' and their perceptions of their post‐telemedicine administrative burden.

The two most common means by which clinicians obtained blood tests following telemedicine consultations were from primary care (40%) or patients’ local hospitals (37%) (Figure 1). Sixty‐three percent of clinicians never or rarely experienced difficulties obtaining blood tests after telemedicine consultations. Interestingly, 100% of clinicians who experienced difficulties indicated that these always involved obtaining micronutrient blood tests. Surprisingly, only 2% of clinicians had attempted to bolster their consultations using surrogate physical examinations such as the sit‐to‐stand test^14^ or SARC‐F test.^15^ Similarly, only 24% of clinicians had attempted to use remote monitoring tools, such as “My PN Tracker.”^16^ After telemedicine consultations, the majority of clinicians stated that the amount of administrative work, such as dictating letters and chasing investigative results, generated was roughly the same as that generated during face‐to‐face consultations (Figure 2). However, rather interestingly, more clinicians (26%) stated that more or much more administration was generated following telemedicine consultations than those who selected the options less or much less (11%).

Sixty percent of clinicians had experienced technical difficulties during telemedicine consultations, with the most common difficulty being a poor quality connection.

The majority of clinicians (73%) felt most or all of their patients were accepting of being reviewed using telemedicine. However, 70% of clinicians stated that they were concerned that telemedicine consultations may not be accessible to the most vulnerable patients they care for (Table 8).

**Table 8:** Clinicians' perceptions regarding preparation for and conduct of telemedicine HPN IF consultations.

	*N*	Yes, %	No, %
Have you received formal training in telemedicine consultations?			
Total	55	5	95
Europe	37	3	97
Australia/NZ	13	15	85
North America	5	0	100
Have you experienced technical difficulties during telemedicine consultations?			
Total	82	60	40
Europe	57	63	27
Australia/NZ	17	65	35
North America	8	25	75
Have you used any form of surrogate physical examination to aid you during telemedicine consultations (eg, sit‐to‐stand test, etc)?			
Total	54	2	98
Europe	37	3	97
Australia/NZ	12	0	100
North America	5	0	100
Have you used any form of remote monitoring technology to aide you during telemedicine consultations (eg, apps such as “my PN tracker,” etc)?			
Total	54	24	76
Europe	37	27	73
Australia/NZ	12	17	83
North America	5	20	80
Do you have any concerns regarding equitable access of vulnerable patient groups to telemedicine consultations?			
Total	54	70	30
Europe	37	70	30
Australia/NZ	12	67	33
North America	5	80	20

Abbreviations: HPN, home parenteral nutrition; IF, intestinal failure; NZ, New Zealand.

When asked what they found most useful about telemedicine consultations, the most common response from clinicians was that they were convenient for their patients. Conversely, when asked what they would improve, common responses included access to technical support to ensure problems that do occur can be fixed and improved device connectivity. Concerningly, 12% of clinicians stated that problems with reimbursement affected their willingness to perform telemedicine consultations, with one clinician stating that telemedicine consultations by specialist nurses were not reimbursed. Most clinicians (84%) were very or somewhat satisfied with telemedicine in the post pandemic period, with 91% stating they would like to continue to provide a mixture of telemedicine and face‐to‐face consultations to their patients and 94% stating that, going forward, their IF services intended to provide ≥25% of their consultations via telemedicine (Table 4).

## DISCUSSION

This study is the first to comprehensively assess the attitudes of patients with IF towards telephone and video consultations, as well as the first to assess the attitudes of members of the IF multidisciplinary clinical team to telemedicine. Our findings clearly show that, although most patients with IF and the clinicians who care for them are satisfied with telemedicine consultations, the majority of patients and clinicians do not feel that telephone and video consultations are of the same standard as face‐to‐face consultations. Furthermore, although both groups felt that telemedicine was a convenient method for conducting consultations and patients felt telemedicine consultations made them feel safer in light of the continuing risk posed by COVID‐19, neither group wanted the majority of their consultations to be conducted via telemedicine.

Interestingly, most clinicians indicated that they would want to receive training in telemedicine or would have wanted to be offered training when they first began assessing patients remotely. This finding is concerning and suggests there is an unmet need for telemedicine training in IF. This is particularly the case for clinicians at the start of their telemedicine journey. In the literature, there have been suggestions that a formal process of training should be used to ensure clinicians are fully equipped to provide telemedicine consultations.^17^ However, it must be noted that there is little evidence in support of the effectiveness of telemedicine consultation training courses in improving consultation skills.^18^, ^19^

The majority of patients with IF had received telemedicine in the form of telephone consultations. This is in agreement with the literature, where it is known that telephone consultations are often the most common means through which telemedicine is provided.^20^ However, the type of telemedicine provided varies between healthcare systems and can be influenced by reimbursement considerations.^21^ Patients expressed a moderate to high degree of satisfaction with all aspects of telephone consultations, agreeing that they were able to easily hear their clinicians, their conversations were appropriately private, they did not feel rushed, they were able to speak about the issues that were important to them, and they were comfortable during their consultations. The majority of clinicians were satisfied with their ability to establish therapeutic relationships with their patients and meet most of their patients’ requirements over the telephone. Although, as stated earlier, no studies have been published which investigate patient attitudes towards telephone consultations in IF, the study findings are in keeping with the high satisfaction with telephone consultations expressed in studies in other medical specialties.^22^, ^23^ Furthermore, the study findings are in keeping with best practice suggestions for telephone consultations that state that, as a minimum, patients must be able to clearly hear their clinicians and must be able to converse in private.^24^ The attitudes of both patients and clinicians towards video consultations mirrored those for telephone consultations, with similarly high satisfaction across most questions. Our study findings echo the similarly high patient satisfaction findings of 85% in the 2020 study by Cloutier et al.^13^

Comparing the pattern of responses between the two telemedicine modalities, clinicians in all geographic regions were highly satisfied with telemedicine, with greater satisfaction with video consultations than with telephone consultations. Additionally, the data show that significantly more clinicians felt better able to meet the needs of their patients using video consultations. When asked to volunteer reasons why they prefer video consultations over telephone consultations, clinicians stated that being able to visualize their patient and, in some cases, their HPN line and surrounding skin enhanced their ability to provide care. In contrast, patient attitudes towards video consultations were mixed (Tables 1 and 2). Examples of this include fewer patients feeling their video consultations were as comfortable and private as telephone consultations. The reasons for this difference are unclear but may reflect the fact that, because of the portability of most telephones, patients can move in search of the privacy and comfort they desire, whether this is in a work or home setting. Conversely, the computer equipment needed for video consultations results in a reduction in portability which may mean absolute privacy may be harder to ensure. Another potential cause for these mixed results is the much smaller number of patients who had received video consultations compared with telephone consultations, meaning small changes in satisfaction were possibly amplified in percentage terms. Interestingly, despite most patients and clinicians being satisfied with video and telephone consultations, the vast majority of patients and clinicians did not feel that telemedicine consultations were of the same standard as face‐to‐face consultations. This potentially explains the high degree of patient and clinician support for hybrid IF consultations observed in our study. It suggests that, although both groups are happy to utilize telemedicine because of its convenience, it is not seen as a substitute for face‐to‐face consultations.

Importantly, most clinicians, in all geographic regions, stated that they currently employed a hybrid consultation model, and, going forward, all clinicians indicated that this will continue. Although patients and clinicians highlighted flexibility and convenience as benefits of telemedicine, neither group stated that they were quicker than face‐to‐face consultations. Furthermore, only a small minority of clinicians stated that telemedicine consultations generated fewer postconsultation administrative tasks than face‐to‐face consultations. Although some studies report that telemedicine is a more efficient means of performing consultations,^6^ other studies have found that clinicians who use telemedicine often do not save administrative time^7^, ^25^ and end up performing more administrative tasks, such as chasing investigative results and drafting letters, outside their contracted hours.^26^ Telemedicine can therefore be viewed as a tool whose primary benefits are the enhancement of flexibility and convenience for patients, but that should not be viewed as being guaranteed to reduce clinician workload or remove the need for face‐to‐face consultations. Unfortunately, despite this, our findings show that, for a substantial minority of clinicians, there has been a reduction in the clinic or office space provided for face‐to‐face consultations since the introduction of telemedicine. For the clinicians affected, the change appears to have been imposed rather than collaboratively introduced, as evidenced by their strong dislike of the reductions and the statements from some clinicians that, since the introduction of telemedicine, there has been increased pressure to see more patients more quickly.

Despite clinicians’ high degree of satisfaction with telemedicine, most were concerned about vulnerable patients being able to equitably access telephone and video consultations. This was the case in all geographic regions studied and suggests the presence of a widespread problem. Studies in other medical specialties have shown that older or less technologically skilled patients are less willing to engage with telemedicine in general, and video consultations in particular.^27^, ^28^, ^29^, ^30^ Ways of addressing this issue that have been suggested in the literature include, only offering video consultations to patients who state they are comfortable and able to use computers and using simplified computer displays and applications.^30^ Despite this no age related differences in satisfaction were observed in this study. Regarding travel distances, as in the earlier Cloutier et al study,^13^ our data showed that patients travelled significant distances to the IF clinic to be reviewed face‐to‐face. The use of telemedicine, therefore, reduces the need for patient travel leading to a reduction in costs and environmental impact.^31^ Therefore, an argument may be made that, although older non technologically skilled patients may be relatively disadvantaged by telemedicine, patients who are technologically skilled but geographically distant from their IF center may be comparatively advantaged. This suggests that a hybrid consultation approach, as currently practiced by survey respondents, is best suited to balancing the opposing issues as regards equity.

After telemedicine consultations, clinicians mostly obtained patient blood tests that were either drawn in their patients’ local hospitals or primary care. Although only a small number of clinicians experienced frequent problems obtaining blood tests, all of those clinicians stated that they had difficulty obtaining micronutrient blood tests. Micronutrient blood tests often require specialized methods of sample transport and assays,^32^ so this finding was perhaps unsurprising. However, this is a problem as monitoring micronutrient levels is required to comprehensively review patients with IF. If there is a consistent issue with obtaining required blood tests because of logistical or other concerns, it can be argued that it is not appropriate to use telemedicine consultations in those settings.

Surprisingly, despite the majority of clinicians stating that being unable to examine patients during telemedicine consultations was a drawback, very few made use of surrogate means of assessing their patients’ physical statuses. Several tools exist that have been validated in other patient groups that allow clinicians to remotely assess patients’ muscle strength and physical dependence.^33^ For example, the SARC‐F and Rockwood clinical frailty tools have recently been shown to be of some use in assessing sarcopenia in patients with type 2 IF.^15^ More work is needed in this area to validate existing tools in IF or develop new more IF focused surrogate tools. These findings were mirrored regarding the use of remote monitoring technology during telemedicine consultations, with only a minority of clinicians making use of applications such as “My PN Tracker.” This is interesting as the lack of widespread use of existing remote monitoring technology and surrogate physical examination tools suggests that there is no concerted effort being made to address the known weaknesses of telemedicine consultations.

Despite the novel nature of this study and its useful findings, several limitations must be borne in mind. Firstly, the patient and clinician surveys were entirely voluntary, which leaves them at risk of selection bias. However, in the patient study, over 20% of the patients in the UK national IF reference center were surveyed, which is a considerable proportion and reduces the risk of unrepresentative findings. Additionally, patient and clinician attitudes towards telemedicine are subjective by their very nature. Without the use and analysis of objective outcome measures^21^ (eg, trends of virtual examination findings compared with in‐person examination findings) and readmission and IF complication rates between telemedicine and face‐to‐face patient populations, it must be acknowledged that our study data forms only a part of the picture and must be interpreted accordingly. Studies incorporating objective outcome measures will need to be conducted in the future.

In conclusion, this is the first study on a national or international level that has assessed patient and clinician attitudes toward telephone and video consultations in IF. Our findings show that, despite a high degree of satisfaction with both telemedicine modalities and the acknowledgement of their convenience, patients and clinicians do not consider telemedicine consultations to be of the same standard as face‐to‐face consultations and would prefer a hybrid model of IF consultations going forward. Unfortunately, most clinicians did not utilize surrogate physical examination tools or means of monitoring patients’ PN remotely. This will need to change in the future to improve the quality of telemedicine consultations. Lastly, and rather concerningly, most clinicians had not received telemedicine training despite feeling it would be useful, revealing a large unmet need in IF services.

## AUTHOR CONTRIBUTIONS

Ayodele Sasegbon designed the study as well as collected, analysed, and interpreted the data before drafting the manuscript. Amy Woods and Francesca Vourloitis helped collect data for the study. Maria Barrett helped design the initial versions of the staff and patient questionnaires. Cristina Cuerda, Palle B. Jeppesen, Francisca Joly, Georg Lamprecht, Manpreet Mundi, Kinga Szczepanek, Andre Van Gossum, Geert Wanten, Tim Vanuytsel, and Loris Pironi reviewed the clinician questionnaire. Simon Lal conceptualized and supervised the study, helped with data interpretation and critically reviewed the manuscript. All authors made contributions to the manuscript and jointly submitted it for publication.

## CONFLICT OF INTEREST STATEMENT

None declared.
